# Reply to Letter: factors affecting COVID-19 outcomes in patients with congenital heart disease

**DOI:** 10.1017/S1047951120004941

**Published:** 2021-01-07

**Authors:** Hammad Jeilani, Bilal I. A. Alriyahi, Abdulla Tarmahomed, Amer Harky

**Affiliations:** 1 Barts and The London School of Medicine and Dentistry, Queen Mary University of London, London, UK; 2 Institute of Medical and Biomedical Education, St George's University of London, London, UK; 3 Department of Cardiology, Alder Hey Children’s Hospital, Liverpool, UK; 4 Department of Cardiothoracic Surgery, Liverpool Heart and Chest, Liverpool, UK; 5 Liverpool Centre for Cardiovascular Science, University of Liverpool and Liverpool Heart and Chest Hospital, Liverpool, UK; 6 Department of Cardiac Surgery, Alder Hey Children’s Hospital, Liverpool, UK

**Keywords:** COVID-19, coronavirus, congenital heart, paediatric patients

In our systematic review of current published literature, we have reported outcomes on 12 papers; 5 of them were case reports, 4 case series, and 3 cross-sectional studies totalling 143 patients, of which 99 patients had CHD.^1^ Based on the available evidence from these studies, we concluded that CHD may increase the risk of poor outcomes for those who are contracting COVID-19; and yet due to limited cohort size and available data, there is a necessity for more research with larger sample sizes in order to have a more justified conclusion. The letter by Ahmad et al^2^ focuses on the fact that differences in age group, race, and complexity of the CHD could be confounders. The authors state that recent evidence suggests that CHD alone is an insufficient indicator of the disease course of COVID-19 and they propose that cardiac markers should be used in conjunction with CHD severity to better predict COVID-19 prognosis, and these should be analysed to mitigate the effect of other highlighted confounding variables such as non-cardiac comorbidities.^2^ While we do understand their comments and agree that patients with CHD may have other medical comorbidities, which can contribute to their incremental risk of COVID-19, and therefore need particular attention, it should be reiterated that CHD and COVID-19-related evidence are emerging and there is a daily change in published literature.^3,4^ In one of our studies, we have reported that COVID-19 is a disease that targets multiple organs, and ethnicity and race can be an important independent contributing factor towards worse outcomes in COVID-19 patients.^5,6^ If this is the case, this in itself would not negate CHD from being a contributory factor.

Ogunjimi et al^3^ recently reported that although there is a scarcity of evidence, patients with CHD are prone to an increased risk of poor outcomes if infected with COVID-19, with the paediatric population being mostly spared. Additionally, given the lack of a large data series, it is currently not possible to perform a meta-regression analysis or identify a single predicting factor for the incremental risk in CHD towards COVID-19-related complications.

In specific response to their cited article by Lewis et al, the results of which they claim to be contrary to our findings, we proffer that a single study cannot be used to generalise outcomes over a large population of CHD patients.^7^ Furthermore, their finding of lacking the correlation between the complexity of CHD and the severity of infection-related cardiac decompensation does not disqualify CHD from being a predictor of worse prognosis in itself. This view is also supported by the conclusions drawn by Tan et al^8^ that patients with heart disease are likely to be at higher risk due to impaired functional reserve, and that late death seen in patients with COVID-19 may be attributable to myocardial damage with resultant cardiovascular collapse.

Overall, we feel there remains an unmet need for larger and multicentre studies with better control of confounding factors to understand CHD and the related COVID-19 incremental risks in all age groups and different pathologies. Fortunately, the British Congenital Cardiac Association is conducting a nationwide, multicentre survey to gauge the impact of COVID-19 on CHD more comprehensively.^9^ We hope this will allow us to better understand the relationship, and therefore provide better preventative strategies for our patients.
